# CP110 and its network of partners coordinately regulate cilia assembly

**DOI:** 10.1186/2046-2530-2-9

**Published:** 2013-07-26

**Authors:** William Y Tsang, Brian D Dynlacht

**Affiliations:** 1 Institut de recherches cliniques de Montréal, 110 avenue des Pins Ouest, Montréal, QC H2W 1R7, Canada; 2 Faculté de Médecine, Université de Montréal, Montréal, QC H3C 3J7, Canada; 3 Division of Experimental Medicine, McGill University, Montréal, QC H3A 1A3, Canada; 4 Department of Pathology and Cancer Institute, Smilow Research Center, New York University School of Medicine, New York, NY 10016, USA

**Keywords:** Centrosomes, Cilia, Ciliogenesis, CP110, Cep290, BBSome, IFT, Protein network

## Abstract

Cilia are hair-like protrusions found at the surface of most eukaryotic cells. They can be divided into two types, motile and non-motile. Motile cilia are found in a restricted number of cell types, are generally present in large numbers, and beat in a coordinated fashion to generate fluid flow or locomotion. Non-motile or primary cilia, on the other hand, are detected in many different cell types, appear once per cell, and primarily function to transmit signals from the extracellular milieu to the cell nucleus. Defects in cilia formation, function, or maintenance are known to cause a bewildering set of human diseases, or ciliopathies, typified by retinal degeneration, renal failure and cystic kidneys, obesity, liver dysfunction, and neurological disorders. A common denominator between motile and primary cilia is their structural similarity, as both types of cilia are composed of an axoneme, the ciliary backbone that is made up of microtubules emanating from a mother centriole/basal body anchored to the cell membrane, surrounded by a ciliary membrane continuous with the plasma membrane. This structural similarity is indicative of a universal mechanism of cilia assembly involving a common set of molecular players and a sophisticated, highly regulated series of molecular events. In this review, we will mainly focus on recent advances in our understanding of the regulatory mechanisms underlying cilia assembly, with special attention paid to the centriolar protein, CP110, its interacting partner Cep290, and the various downstream molecular players and events leading to intraflagellar transport (IFT), a process that mediates the bidirectional movement of protein cargos along the axoneme and that is essential for cilia formation and maintenance.

## Review

### Links between cilia, centrosomes, and the cell cycle

It is well known that cilia and centrosomes share an intimate relationship during the cell cycle. A centrosome consists of a pair of centrioles, termed the mother and daughter centrioles, embedded in a poorly defined pericentriolar matrix, from which cytoplasmic microtubules emanate and grow [1-4]. The mother centriole can be distinguished from the daughter centriole by the presence of distal and sub-distal appendages. Distal appendages are thought to be important for the docking of a basal body to the cell membrane and the recruitment of IFT proteins prior to cilia assembly, whereas sub-distal appendages anchor microtubules, participate in endosome recycling, and form the basal foot, a structure essential for ciliogenesis and ciliary beating in motile cilia [5-9]. In proliferating cells, a single centrosome in the G1 phase undergoes duplication in the S phase. The two centrosomes then separate, migrating to opposite poles and establishing a bipolar spindle in mitosis. Upon cell cycle exit, a centrosome obtains competence for ciliogenesis, whereby the mother centriole is converted into the basal body. Depending on the cell type and/or cilia type, the basal body can migrate and anchor to the cell surface or dock ciliary vesicles, which elongate and eventually fuse with the plasma membrane. In both scenarios, the basal body serves to nucleate the growth of axonemal microtubules, a process highly dependent on IFT [10-12]. IFT is bidirectional, and this property can be explained by the existence of biochemically and functionally distinct protein complexes, IFT-B and IFT-A. While IFT-B and IFT-A are commonly believed to direct anterograde (cell body to cilia) and retrograde (cilia to cell body) transport of macromolecules, respectively, recent evidence indicates that IFT-A is also involved in anterograde transport [13-16]. IFT is essential for cilium assembly and maintenance, since the organelle lacks protein synthesis machinery [17]. When cells re-enter the cell cycle, cilia are disassembled, and the basal body relocates to the cell interior, assuming a position near the nucleus. It is logical to postulate that controls must exist to suppress the inappropriate assembly of cilia in proliferating cells or the untimely assembly of a bipolar spindle in non-proliferating cells. In addition, vesicular trafficking, mother centriole/basal body migration to the cell surface, basal body anchoring to the cell membrane, and IFT must be tightly regulated in a temporally-, spatially-, and cell-type-specific manner to ensure the fidelity of ciliogenesis. Indeed, a growing number of proteins, including many that were originally identified in a proteomic screen for novel centrosomal and ciliary components [18-20], are known to modulate cilia assembly in a positive or negative manner [21,22], suggesting that cilia assembly involves a complex circuitry controlled by the coordinated inhibition of negative regulators and recruitment and activation of positive regulators.

### The CP110-Cep97 pathway

**Figure 1:**
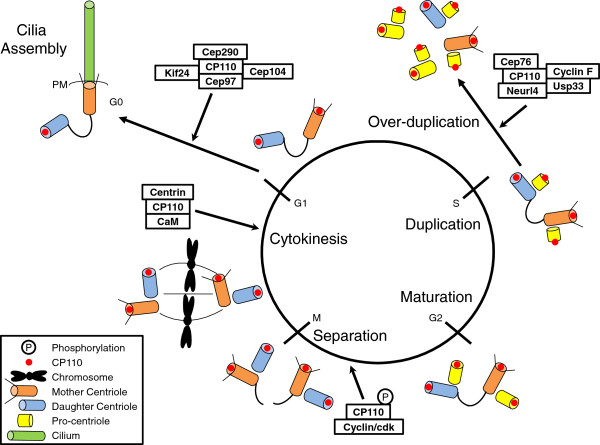
**The role of CP110 in cell cycle control and ciliogenesis.** CP110 and its network of partners form distinct complexes that regulate different aspects of centrosome function, including centrosome over-duplication, centrosome separation, cytokinesis, and cilia assembly. The localization of CP110 is also illustrated. PM denotes plasma membrane.

While there are many important modulators of ciliogenesis, two distal centriolar proteins, CP110 and Cep97, were the first proteins shown to negatively regulate cilia assembly [23]. Loss of either protein elicits premature inappropriate cilium formation in proliferating cells, whereas its over-expression inhibits ciliogenesis in non-proliferating cells. Fittingly, patients with chronic rhinosinusitis, a respiratory disease associated with abnormal or lack of motile cilia, have elevated levels of CP110 [24]. While the precise function of Cep97 awaits further experimentation, this protein might serve as a chaperone to stabilize CP110, allowing the co-recruitment of both proteins to the centrosome. CP110, on the other hand, is thought to impose a structural role at the centrosome and forms discrete complexes critical for cell cycle regulation and cilia assembly (Figure 1) [23,25-31]. This protein does not have an associated enzymatic activity; rather, it was shown to localize to the distal ends of centrioles, forming a ‘cap’ above the growing microtubules that could restrain microtubule growth [32]. Indeed, CP110 has the ability to control centriole length in non-ciliated human [33-35] and insect cells [36] and to block ciliary axoneme formation in ciliated mammalian (RPE-1 and NIH-3T3) cells [23,25]. Paradoxically, CP110 does not modulate cilia length, suggesting that at least in ciliated cells, CP110 could ‘switch off’ the ciliogenic program. Tellingly, CP110 is completely extinguished from the basal body in ciliated cells (Figure 1 and [23]). The loss of CP110 effectively liberates the mother centriole from its centrosomal role in cell division and ‘licenses’ the transition from mother centriole to basal body. Thus, it appears that the removal of CP110 from the mother centriole, rather than cell cycle control *per se*, could play a crucial role in the initiation of ciliogenesis.

**Figure 2:**
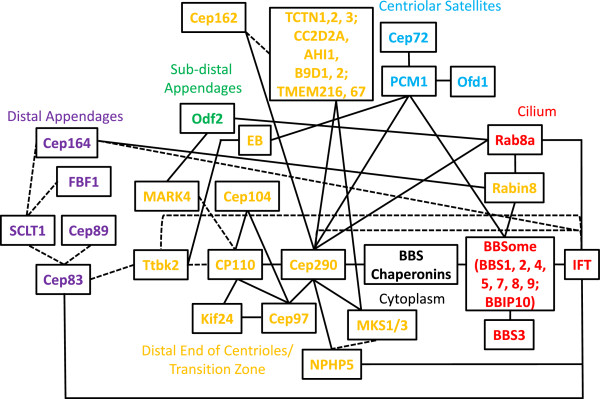
**A system-wide schematic of protein interaction networks that modulate cilium assembly.** Solid lines indicate known protein-protein interactions, confirmed by immunoprecipitation, yeast two-hybrid, and/or *in vitro* binding experiments. Not every protein-protein interaction indicated is direct. Dashed lines indicate known functional connections with no evidence of protein-protein interactions to date. EB denotes end binding proteins.

CP110 levels and localization to the centrosome are tightly regulated in a cell cycle dependent manner [29]. CP110 protein levels drop significantly in G2/M and G0/G1 phases as a consequence of transcriptional controls, ubiquitin-mediated proteasomal destruction, and microRNA-mediated turnover of CP110 mRNA [37-39]. Furthermore, disappearance of CP110 from the basal body in quiescent cells coincides with an enrichment of a serine/threonine kinase, Ttbk2, at the same location (Figure 2) [40]. Ttbk2, a microtubule plus-end tracking protein, likely promotes the onset of ciliogenesis by cooperating with end binding proteins [40-42]. Depletion of Ttbk2 impairs both the loss of CP110 and the recruitment of IFT complexes, including IFT88, a protein localized to the distal appendages of the emerging basal body and/or the transition zone [40]. Further, the loss of Cep83, a distal appendage protein that functions in a concerted and hierarchical manner to recruit other proteins (including Cep89, SCLT1, FBF1, and Cep164), prevents the recruitment of Ttbk2 to, and the release of CP110 from, the basal body, thereby blocking basal body anchoring to the cell membrane (Figure 2) [43]. Another study highlighted a role for CCDC41/Cep83 in the recruitment of IFT20 to the basal body and ciliary vesicle docking to the mother centriole as important functions of CCDC41/Cep83 during early ciliogenesis, although Cep164 localization and abundance were not substantially impacted [44]. Since Cep83 and Cep164 can recruit IFT proteins to the basal body and/or the transition zone, these results imply that distal appendage proteins, Ttbk2, CP110, and IFT proteins could functionally interact [43,45]. In addition to Ttbk2, the loss of a second serine/threonine kinase, MARK4, causes mis-localization of its interacting partner, Odf2, which is normally found at sub-distal appendages, and likewise, inhibits cilia formation by preventing the removal of CP110/Cep97 from the basal body (Figure 2) [46-48]. In light of recent findings that distal and sub-distal appendages are assembled independently of one another [43], these intriguing observations suggest that Ttbk2 and MARK4 activities might be necessary to modulate the molecular framework of distal and sub-distal appendages, respectively, ultimately leading to the destruction and removal of CP110 from the basal body. Alternatively, the two kinases could function after the assembly of the appendages to remove CP110 [49]. Furthermore, these studies suggest that protein phosphorylation is crucial for the maturation of a mother centriole into a functional basal body, and future phospho-proteomic studies, in combination with high resolution imaging, will be essential to identify key substrates and to examine these maturation events in greater detail.

### CP110-interacting partners and its protein network

Besides Cep97, CP110 has been shown to associate with a cadre of proteins important for ciliogenesis, suggesting that it could assemble a multi-functional platform to integrate centriolar and basal body functions (Figure 2). Cep104, a microtubule plus-end tracking protein identified by a proteomic screen for novel end binding- interacting partners, interacts with CP110 and Cep97 [41]. This protein co-localizes with CP110 at the distal ends of centrioles in proliferating cells and is similarly absent from the basal body in quiescent cells. However, unlike CP110 and Cep97, Cep104 is essential for ciliogenesis, suggesting that it may regulate axonemal growth at the onset of cilia assembly by counteracting the activities of CP110 and Cep97. In contrast to Cep104, another protein, Kif24, appears to reinforce the role of CP110 as a suppressor of ciliogenesis [28]. As a member of the microtubule de-polymerizing kinesin family of proteins, Kif24 specifically de-polymerizes and remodels centriolar microtubules at the mother centriole/basal body, and depletion of this protein promotes ciliation, whereas over-expression inhibits cilia growth. Although Kif24 binds CP110 and Cep97, it specifically stabilizes CP110 and recruits it to the centrosome, suggesting that both the de-polymerizing activity of Kif24 and its ability to recruit a distal end capping protein (CP110) to centrioles contribute to cilia suppression. In addition, CP110 has been demonstrated to associate with a human ciliopathy protein, Cep290, (also known as BBS14, NPHP6, JBTS5, SLSN6, MKS4 and LCA10; [25]). Its many names can be attributed to the diverse spectrum of clinical manifestations, including Bardet-Biedl syndrome (BBS), nephronophthisis, Joubert syndrome, Senior-Loken syndrome, Meckel-Gruber syndrome, and Leber congenital amaurosis, associated with mutations in the *Cep290* gene [50-52]. Despite the identification of over 100 unique mutations, there is no clear relationship between genotype and phenotype. The loss of Cep290 abolishes cilia assembly and disrupts the migration/anchoring of centrioles to the cell cortex, suggesting that this protein functions to promote ciliogenesis at an early step of the ciliogenic pathway [21,25,53]. This positive function of Cep290 is antagonized by CP110, and over-expression of a CP110 mutant refractory to Cep290 binding is incapable of suppressing ciliation in non-proliferating cells. Because the protein levels of Cep290 remain constant throughout the cell cycle, including G0 [25], it seems plausible that CP110 restrains Cep290 activity in proliferating cells through direct interaction, but once cells exit the cell cycle, the loss of CP110 protein releases Cep290 from inhibition. It is currently not clear how Cep290 might promote centriole migration/anchoring to the cell cortex, although it is known that this protein directly interacts with another ciliopathy protein NPHP5 [54], and depletion of NPHP5 phenocopies the loss of Cep290 [55-57]. Interestingly, analysis of the primary amino acid sequence of Cep290 reveals the presence of multiple N-terminal tropomyosin homology domains and a C-terminal myosin-tail homology domain, suggesting that it might have an actin-related function, and that centriole migration/anchoring could involve cytoskeletal re-organization and modulation of actin dynamics [51,58]. Indeed, the role of actin cytoskeleton dynamics in cilia assembly has recently been illustrated in a high-throughput RNA interference screen, wherein actin polymerization was shown to have an inhibitory role in cilia assembly [22]. Two proteins belonging to the gelsolin family members, GSN and AVIL, promote ciliation by severing actin filaments. On the other hand, ACTR3, a protein known to mediate the formation of branched actin networks, suppresses cilia formation. Treatment of cells with drugs that inhibit actin filament polymerization and/or affect actin dynamics, such as cytochalasin D or latrunculin B, can facilitate ciliation in addition to causing an increase in cilium length [22,55]. Notably, impaired cilia formation associated with the loss of Cep290 or NPHP5 can be restored by the aforementioned drugs, strongly suggesting that proteins involved in the regulation of actin dynamics could influence the ciliogenic pathway and could be exploited as potential therapeutic targets [55]. Besides Cep290 and NPHP5, two other ciliopathy-associated proteins, MKS1 and MKS3, are also required for the translocation of centrioles to the cell surface, whereas IFT88 is not [59]. Thus, it seems likely that a subset of centrosomal proteins is specifically dedicated to basal body migration and anchoring to the cell membrane, and it will be most interesting to identify the complete set of factors that control this important process.

### Cep290 function and its protein network

Beyond its potential contribution in basal body migration and/or anchoring to the cell membrane, Cep290 has additional functions critical to cilia assembly. An elegant ultra-structural study conducted in *Chlamydomonas reinhardtii* suggests that Cep290 localizes to the transition zone, a small region immediately distal to the basal body characterized by the presence of Y-shaped fibers that connect the axonemal microtubules to the ciliary membrane [60]. This region is thought to regulate the entry and exit of protein and lipid cargos into and out of the ciliary compartment. Consistent with this idea, Cep290 is present at the transition zone of rat motile tracheal cilia [61] and associates with CC2D2A and TCTN1, both of which are known to form a large protein complex with several other ciliopathy proteins (AHI1, MKS1, TCTN2, TCTN3, B9D1, B9D2, TMEM216, TMEM67) at the transition zone (Figure 2 and [62-64]). Cep290 also binds to Cep162, an axoneme-recognition protein required for transition zone assembly (Figure 2 and [65]). In addition, Cep290 is required for the targeting of Rab8a, a small GTPase responsible for vesicular trafficking into the cilium in cultured human epithelial cells [25,53], and has a functional connection with the BBSome, a stable multi-subunit complex known to mediate ciliary transport (Figure 2). The BBSome is composed of seven BBS proteins (BBS1, BBS2, BBS4, BBS5, BBS7, BBS8, and BBS9) and BBIP10, a protein required for cytoplasmic microtubule polymerization and acetylation. Assembly of the BBSome follows a hierarchical order that initially involves the stabilization of BBS7 by the chaperonin complex (MKKS/BBS6, BBS10, BBS12 and CCT/TRiC family of chaperonins), followed by the formation of the BBSome core (BBS7, BBS2, BBS9) and the subsequent incorporation of the remaining BBSome subunits through a series of protein-protein interactions [66,67]. Interestingly, two components of the BBSome, BBS4 and BBS8, are not properly recruited to the cilium upon Cep290 loss [68]. The lack of BBSome recruitment to the cilium could be due to an assembly defect, as Cep290 is known to directly interact with MKKS/BBS6, a chaperonin-like molecule required at an early step in BBSome assembly [69]. In addition, a Cep290 mutant in *Chlamydomonas reinhardtii* possesses malformed flagella with abnormal protein composition, with increased amounts of IFT-B proteins and decreased amounts of IFT-A proteins, suggesting that retrograde and possibly anterograde IFT are impaired [60]. Although neither Cep290 nor CP110 has been demonstrated to directly interact with IFT proteins thus far, a proteomic screen reveals IFT122 as a novel interacting partner of NPHP5 (Figure 2 and [56]), a protein that directly binds to, and shares a number of common features with, Cep290 [55-57]. Further experiments will be necessary to delineate the extent to which the CP110-Cep290 axis overlaps with the BBSome and/or the IFT pathway.

Other than its localization to the transition zone, Cep290 is also targeted to centriolar satellites [53,58]. Centriolar satellites are small, electron-dense proteinaceous granules found in the vicinity of the centrosome and have been implicated in microtubule-dependent protein trafficking towards the centrosome [70-72]. These structures may be closely related to the pericentrosomal pre-ciliary compartment reported at the basal body during the onset of ciliogenesis [22]. Interestingly, several satellite components, including PCM1, BBS4, OFD1, Cep72, and Cep290 are required for cilia assembly, and the integrity of these unique structures is highly dependent on protein-protein interactions between them (Figure 2) [53,68,73]. Of note, BBS4 is unique among satellite proteins in that it completely re-localizes from its original satellite position to the cilium during ciliogenesis [74]. Thus, Cep290, together with other satellite proteins, might regulate the trafficking of BBS4 between the two different sub-cellular compartments, and hence play an indirect role in BBSome assembly. Further studies will be needed to decipher the mechanisms through which satellite proteins (and possibly other unidentified associated factors) modulate the number, size, and integrity of satellites in space and time and how such modulation contributes to basal body function, transition zone assembly, and ciliogenesis.

### The role of the BBSome and the IFT complex

BBS is a ciliopathy characterized by renal and retinal failure, obesity, polydactyly, diabetes, hypogenitalism, and hypertension [75]. Seventeen causative genes have been identified so far, and recent studies have begun to unravel the role of BBS proteins in cilia homeostasis. As mentioned earlier, eight different proteins (BBS1, BBS2, BBS4, BBS5, BBS7, BBS8, BBS9, and BBIP10) are required to form a functional unit called the BBSome [74,76]. Intriguingly, the BBSome binds Rabin8, a GDP/GTP exchange factor for Rab8a, and directly interacts with phospholipids, suggesting that this complex likely mediates vesicular trafficking during ciliogenesis (Figure 2) [74]. More recently, another BBS subunit, BBS3/Arl6, an Arf-like GTPase, was shown to be a major effector of the BBSome [77]. BBS3/Arl6 recruits the BBSome to the membrane, where it assembles a ‘coat’ that sorts proteins to the cilium. This ‘coat’ recognizes a unique ciliary localization signal found in several ciliary membrane proteins, leading to their efficient trafficking to the cilium [77,78]. Future biochemical and biophysical studies will shed light on the structure of the ‘coat’ and the precise nature of the ciliary localization signal it recognizes.

Although the BBSome is thought to play an important role in sorting certain membrane proteins to the cilium, neither this complex, nor its assembly factors or BBS3/Arl6, is generally required for ciliogenesis, as depletion or loss of some of these proteins does not severely impair ciliation but rather leads to defective IFT transport [79-82]. In addition, while BBS knockout mice (BBS1, BBS2, BBS4, BBS6 or BBS7) display subtle phenotypes [81,83-86], a loss of BBS7 in combination with a reduction in IFT function results in a more severe phenotype [85], suggesting that the BBSome and the IFT complex could function in a synergistic manner. These findings have led to the speculation that the BBSome is only responsible for transporting a subset of ciliary proteins, whereas the IFT complex is more universally required for all transport processes. Recently, an elegant study which combines a whole-genome mutagenesis screen for mutants with abnormal cilia formation, time-lapse microscopy, and bimolecular fluorescence complementation in *Caenorhabditis elegans* showed that the BBSome acts on the IFT complex by controlling its assembly and turnaround in cilia [14]. The BBSome first interacts with the IFT complex (Figure 2) and motor proteins to organize them into a functional super-complex. This super-complex undergoes anterograde transport to the ciliary tip, and once there, the BBSome dissociates from the IFT complex, unloading cargos during the process. The BBSome then re-organizes the IFT complex and re-loads new cargos for retrograde transport back to the ciliary base. It remains to be determined if the role of the BBSome in worms is mechanistically conserved in higher eukaryotes, since subtle differences exist in the ciliary structures, and not every BBS subunit is evolutionarily conserved. Nevertheless, elucidating the molecular functions of the individual BBS and IFT components would undoubtedly provide a better understanding of how these two complexes coordinately promote cilia assembly.

## Conclusions

Our knowledge of the architecture of the cilium and the functions of individual ciliary components has expanded considerably in the past 10 to 15 years. The use of forward and reverse genetic screens, animal models, system-wide proteomics, time-lapse microscopy, cryo-electron microscopy, and new innovations in super-resolution microscopy have led to rapid and unprecedented breakthroughs in the field, highlighted by many landmark discoveries. Among these, CP110 and Cep290 have emerged as key players in the regulation of the cilia assembly process. Despite our current knowledge of their functions, important questions remain: is CP110 the protein responsible for the conversion of mother centrioles (ciliogenesis incompetent) to basal bodies (ciliogenesis competent), and how are the diverse functions of Cep290 intertwined, if at all, in modulating cilia assembly? We believe that the answers to these questions lie in our ability to decipher and build upon the existing ciliary protein interaction network (Figure 2). These studies should allow us to understand how this network contributes to health and disease and to devise rational therapeutic approaches for treating ciliopathies based on these proteomic and genetic networks.

## Abbreviations

IFT: Intraflagellar transport; BBS: Bardet-Biedl syndrome.

## Competing interests

The authors declare that they have no competing interests.

## Authors’ contributions

WYT wrote the manuscript. WYT and BDD revised the manuscript. Both authors read and approved the final manuscript.
